# Vitamin D deficiency in girls from South Brazil: a cross-sectional study on prevalence and association with vitamin D receptor gene variants

**DOI:** 10.1186/1471-2431-12-62

**Published:** 2012-06-08

**Authors:** Betânia R Santos, Luis P G Mascarenhas, Fabíola Satler, Margaret C S Boguszewski, Poli Mara Spritzer

**Affiliations:** 1 Gynecologic Endocrinology Unit, Division of Endocrinology, Hospital de Clínicas de Porto Alegre (HCPA), Porto Alegre, Brazil. Laboratory of Molecular Endocrinology, Department of Physiology, Universidade Federal do Rio Grande do Sul (UFRGS), Porto Alegre, Brazil; 2 Department of Pediatrics, Universidade Federal do Paraná (UFPR), Curitiba, Brazil; 3 National Institute of Hormones and Women’s Health, Porto Alegre, Brazil

**Keywords:** 25-hydroxyvitamin D, VDR gene polymorphisms, Pediatric female population

## Abstract

**Background:**

Vitamin D deficiency has been associated with a multitude of disorders including diabetes, defective insulin secretion as well as rickets and poor bone health. Vitamin D is also a concern during childhood and adolescence and has been reported in girls from South Brazil. We determined the prevalence of vitamin D deficiency in girls from South Brazil and investigated whether the genotypic distribution of the BsmI, ApaI and TaqI polymorphisms of the VDR gene and their haplotypes were associated with vitamin D levels.

**Methods:**

Cross-sectional study including 234 apparently healthy girls aged 7 to 18 years. Height and weight were measured for calculation of body mass index (BMI) percentiles for age. Plasma levels of 25-hydroxyvitamin D [25(OH)D] were assessed. Participants were genotyped for ApaI (rs7975232), TaqI (rs731236), and BsmI (rs1544410) SNPs.

**Results:**

The median and interquartile range (25-75%) of BMI percentile was 62.0 (33.3 – 84.9). The frequency of overweight/obesity was 24.9%. Circulating levels of 25(OH)D (≥ 30 ng/mL) were adequate in 9.4%; insufficient in 54.3% (20–29 ng/mL); and deficient in 36.3% (< 20 ng/mL). Genotype frequencies were GG = 47.0%, GA = 41.5%, and AA = 11.5% for BsmI; GG = 16.7%, GT = 52.6%, and TT = 30.8% for ApaI; TT = 46.2%, TC = 44.9% and CC = 9.0% for TaqI. Genotypes with no gene variance (ancestral wild genotype) of BsmI (GG *vs.* GA + AA, two-tailed Student’s *t*-test p < 0.001), ApaI (GG *vs.* GT + TT, two-tailed Student’s *t*-test p = 0.031) and TaqI (TT *vs.* TC + CC, two-tailed Student’s *t*-test p = 0.005) SNPs and the GGT haplotype (two-tailed Student’s *t*-test p = 0.036) were significantly associated with lower 25(OH)D levels.

**Conclusions:**

25-hydroxyvitamin D deficiency and insufficiency were highly prevalent in this sample. The BsmI, ApaI and TaqI wild variants of the VDR gene, as well as the GGT haplotype, were associated with lower vitamin D levels, suggesting that VDR gene polymorphisms could be linked to higher susceptibility to vitamin D deficiency in a sub-population of children and adolescents.

## Background

Vitamin D is mainly recognized for its effects on bone metabolism and for being an important determinant of growth and body development during childhood and adolescence. However, a high prevalence of vitamin D deficiency has been observed in several pediatric and adolescent populations [1-3] in association with overweight/obesity, blood pressure and glucose metabolism [4-6]. While these studies report no gender-related differences in vitamin D levels, recent evidence shows that girls have significantly lower vitamin D levels than boys [7]. Our group has also observed a high prevalence of vitamin D deficiency in girls with precocious pubarche and controls with normal pubertal development in South Brazil [8].

The vitamin D receptor (VDR) gene regulates up to 200 genes [9], and mediates most effects of vitamin D on gene expression *via* formation of a heterodimer with the retinoid x receptor, which binds to promoter regions of many target genes [9]. Several polymorphisms have been described for the VDR gene (ID: 7421), located on chromosome 12 (12q13.11), consisting of 11 exons and spanning 63495 bp. BsmI, ApaI (both located in intron 8) and TaqI (located in exon 9) are the most studied variants. VDR gene polymorphisms have been linked with specific health outcomes, including low bone density in postmenopausal women [10], type 2 diabetes or metabolic syndrome [11,12] and low 25-hydroxyvitamin D (25(OH)D) concentration [13,14]. Given the high prevalence of vitamin D deficiency in children and adolescents described earlier, it may be speculated that VDR gene polymorphisms could be linked to higher susceptibility to develop vitamin D deficiency.

Therefore, the aims of the present study were to assess the genotypic distribution of the BsmI, ApaI and TaqI polymorphisms of the VDR gene in a population of girls from South Brazil and to determine whether these gene variants and their haplotypes are associated with 25(OH)D levels.

## Methods

### Subjects

This cross-sectional study was carried out between April 2008 and January 2011 and included 234 apparently healthy girls aged 7 to 18 years who had parental consent to participate in the study. Two hundred and thirteen girls recruited at four public schools from the four main regions of the city of Curitiba (North, South, East, and West), in the state of Paraná, Brazil (latitude −25°), and 21 girls enrolled at a vaccination facility or University adolescent clinic in the city of Porto Alegre (latitude −30°), state of Rio Grande do Sul, were included in the study. None of the girls took calcium or vitamin D supplements. Two girls used birth control pills and 19 made occasional use of bronchodilators or nasal corticosteroids for asthma or rhinitis. Most of these girls were also included in the control group of a previous study [8].

Approval for this study was obtained from the Institutional Review Boards and the local Ethics Committees of Hospital de Clínicas de Porto Alegre and Universidade Federal do Paraná. Written informed consent was obtained from all participants or their caretakers.

### Study protocol

All subjects underwent physical examination. On that occasion, information on thelarche and menarche age was collected through interview. Anthropometric measurements included height and weight for subsequent calculation of body mass index (BMI). Height was obtained by using a stadiometer fixed to the wall and weight was obtained by using a digital balance, with resolution of 100 grams. The subjects were evaluated barefoot and wearing light clothing. Individual height and BMI values were converted into percentiles according to age based on 2000 Centers for Disease Control and Prevention charts [15]. For that, the software EpiInfo/AnthropometricData (version 3.5.1) was used.

25(OH)D, the main vitamin D circulating metabolite, was assessed in blood samples drawn between 8:00 and 10:00 AM from an antecubital vein, after an overnight fast. Blood samples were also collected for genomic DNA extraction.

Girls without thelarche at the time of enrollment were defined as prepubertal. Subjects were classified as normal weight (BMI < 85 percentile), overweight (85 percentile ≤ BMI ≤ percentile 95) or obese (BMI > 95 percentile). Serum 25(OH)D status was classified as sufficient (≥ 30 ng/mL), insufficient (20–29 ng/mL) or deficient (< 20 ng/mL).

### Assays

Serum 25(OH)D (sensitivity = 1.5 ng/mL) was measured with radioimmunoassay (DiaSorin, Stillwater, USA) with intra and inter-assay coefficients of variation of <12.0% and < 15.0% respectively.

### Genotype analysis

Genomic DNA was extracted from peripheral blood leukocytes. Molecular genotyping for the ApaI (rs7975232) and TaqI (rs731236) SNPs (change of the G → T and T → C, respectively) was performed by polymerase chain reaction (PCR) followed by restriction fragment length polymorphism (RFLP) analysis [16]. Forward 5’-GTTCAGCAGCAAATGGGACACA-3’ and reverse 5’-AGCTTCTGGATCATCTTGGCATAG-3’, primer sequences yielded a 740 bp PCR product. Protocol conditions consisted of denaturation at 95°C for 2 min followed by 35 cycles (95°C, 30sec; 59.2°C, 30sec; 72°C, 80sec) and final extension at 72°C for 5 min. PCR products were digested overnight by the restriction enzymes ApaI or TaqI (New England Biolabs, USA) at 37°C or 65°C, respectively. ApaI digestion revealed genotypes TT (740 bp), TG (740, 559, and 181 bp) or GG (559 and 181 pb), while TaqI digestion denoted genotypes TT (740 bp), TC (740, 635 and 105 pb) or CC (635 and 105 pb) at 2% agarose gel electrophoresis.

BsmI (rs1544410) SNP (change of the G → A) genotyping was performed through real-time PCR (7500 Fast Applied Biosystems, California, USA) with allelic discrimination assays (Taqman MGB Probes®) according to the manufacturer’s instructions (Applied Biosystems, California, USA).

We calculated Lewontin's D' (|D'|) r^2^ between each pair of genetic markers for estimating the linkage disequilibrium. Haplotypes were constructed from the combination of the three VDR polymorphisms (BsmI, ApaI and TaqI), and their frequencies were inferred using the PHASE 2.1.1 program. The first letter of each haplotype refers to BsmI polymorphism, the second to the ApaI polymorphism, and the third to TaqI polymorphism.

### Statistical analysis

The sample size estimation was based on the study by Bhanushali *et al.*[13], in which a different frequency of the SNP TaqI was found in relation to vitamin D status in adults. Therefore, considering the same difference, an alpha of 5% and a beta of 90%, the sample size was estimated as 228 girls, 94 with genotype TT and 134 with genotypes TC + CC.

Data were described as mean ± standard deviation (SD) (Gaussian variables) or median and interquartile range (25-75%) (non-Gaussian variables). Comparisons between means were analyzed by the unpaired two-tailed Student’s *t*-test or one-way analysis of variance (ANOVA) followed by Tukey’s post hoc test. Log_10_ transformation was used to normalize the distribution of non-Gaussian variables and mean values were back-transformed for presentation. Data were adjusted by season and/or age at the time of blood collection by linear regression.

Categorical variables and the agreement of genotype frequencies with Hardy-Weinberg equilibrium for each SNP were analyzed using the Pearson chi-square test [χ^2^]. Odds ratios (OR) and 95% confidence intervals (95%CI) were obtained using χ^2^ risk estimate. Multiple linear regression analysis models were set up to determine the independent effects of season, age, and BMI percentile for each of the polymorphisms (BsmI, ApaI and TaqI) and the Ht1 haplotype, having plasma 25(OH)D as the dependent variable. Data were considered as statistically significant at p < 0.05. The Statistical Package for the Social Sciences 16 (SPSS, Chicago, IL) was used in the analyses.

## Results

All the 234 healthy girls who were enrolled following parental consent completed the interview, physical examination and blood collection. They were studied during spring/summer [35 (15%)] or fall/winter [199 (85%)]. Mean chronological age was 13.0 ± 1.9 years. The median BMI percentile was 61.9 (33.3 - 84.9). The frequency of overweight was 15.7%, and of obesity 9.2%. Only 4.0% of the participants were prepubertal, and 34.2% had not had menarche.

Table 1 shows clinical features and 25(OH)D levels in girls according to tertiles of age. Height and BMI percentiles were adequate for all age categories. 25(OH)D levels did not differ among age categories. Mean serum 25(OH)D was 21.3 ± 6.8 ng/mL. Stratification by 25(OH)D status revealed sufficient circulating levels (≥ 30 ng/mL) in only 9.4% of the overall group; 54.3% were insufficient (20–29 ng/mL) and 36.3% were deficient (< 20 ng/mL). No associations were found between season at the time of the blood collection and 25(OH)D levels (spring/summer = 21.4 ± 6.7 ng/mL *vs.* fall/winter = 21.3 ± 6.9 ng/mL; p = 0.955). No significant differences were found between season at the time of the blood collection and 25(OH)D status (χ^2^ = 0.249; p = 0.883).

**Table 1:** Anthropometric and clinical features of girls according to tertiles of age

**Variable**	**Age in years (tertile)**			**p**
	**<12.04 (77)**	**12.04-14.05 (80)**	**>14.05 (77)**	
Age (years)	10.9 ± 1.1^a^	13.0 ± 0.6^a^	15.2 ± 1.1^a^	<0.001
*Height percentile	66.21 (39.99-82.14)	55.65 (31.45-74.40)	41.87 (19.75-77.13)	0.066
*BMI percentile	62.9 (31.8-86.4)	59.9 (35.1-85.1)	61.8 (31.7-82.1)	0.967
Age at thelarche (years)	9.9 ± 1.0^a^	10.3 ± 1.1	10.5 ± 1.2^a^	0.013
Age at menarche (years)	10.9 ± 1.2^a^	11.6 ± 1.0^b^	12.1 ± 1.3^ab^	0.002
25(OH)D (ng/mL)	20.77 ± 6.97	22.10 ± 6.01	21.09 ± 7.33	0.492

Values are expressed as mean ± SD or median and interquartile range (25-75%).

p value by one-way analysis of variance (ANOVA).

* Log_10_ transformation.

Equal letters mean statistical difference between groups.

Concerning VDR polymorphisms, all three SNPs (BsmI, ApaI and TaqI) were in Hardy-Weinberg equilibrium. Genotype frequencies were GG = 47.0% (n = 110), GA = 41.5% (n = 97), and AA = 11.5% (n = 27) for BsmI SNP; GG = 16.7% (n = 39), GT = 52.6% (n = 123), and TT = 30.8% (n = 72) for ApaI SNP; TT = 46.2% (n = 108), TC = 44.9% (n = 105), and CC = 9.0% (n = 21) for TaqI SNP. Twenty-one randomly selected samples (around 10%) were genotyped twice, and the reproducibility of genotyping was 100%.

The BsmI (G → A) polymorphism was in partial linkage disequilibrium with the ApaI (G → T) polymorphism (|D’| = 0.964; r^2^ = 0.330), and in almost complete linkage disequilibrium with the TaqI (T → C) polymorphism (|D’| = 0.919; r^2^ = 0.807). The ApaI (G → T) polymorphism was also in partial linkage disequilibrium with the TaqI (T → C) polymorphism (|D’| = 0.970; r^2^ = 0.319). Seven haplotypes were inferred in the sample (Ht1: GGT, Ht2: GGC, Ht3: GTT, Ht4: GTC, Ht5: AGT, Ht6: ATT and Ht7: ATC). Haplotype frequencies were Ht1: 0.419; Ht2: 0.004; Ht3: 0.242; Ht4: 0.013; Ht5: 0.006; Ht6: 0.019 and Ht7: 0.297.

**Figure 1:**
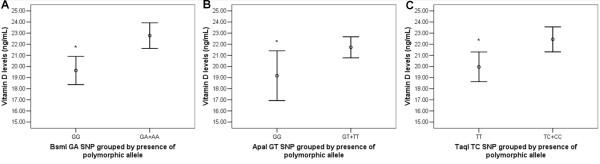
**Vitamin D levels according to BsmI (A), ApaI (B), and TaqI (C) SNPs in VDR gene grouped by presence of polymorphic allele.** Values are expressed as means (central circle) and 95% CI (lower and upper limit). Two-tailed Student’s *t*-test, *p < 0.05 BsmI GG (n = 110) and GA + AA (n = 124); ApaI GG (n = 39) and GT + TT (n = 195); TaqI TT (n = 108) and TC + CC (n = 126).

**Figure 2:**
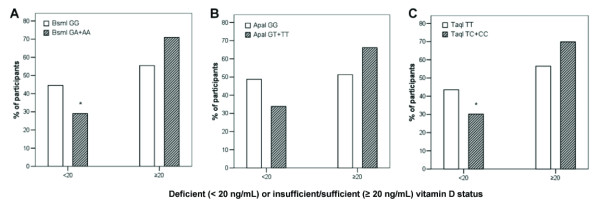
**Frequency of vitamin D deficiency according to BsmI (A), ApaI (B), and TaqI (C) SNPs in VDR gene grouped by presence of polymorphic allele.** White bars express the frequency of vitamin D status *(*deficiency or insufficiency/sufficiency) for the wild genotype of each VDR SNP. Gray bars express the frequency of vitamin D status for the polymorphic (in heterozygous plus homozygous) genotypes. Pearson chi-square test, *p < 0.05.

No differences were found in age, height and BMI percentiles, age at menarche or at thelarche between genotypes for all the three polymorphisms. In contrast, presence of the wild-type genotypes BsmI, ApaI and TaqI SNPs of the VDR gene was significantly associated with lower 25(OH)D levels (BsmI p < 0.001; ApaI p = 0.031; TaqI p = 0.005), even after adjustment for season at the time of blood collection and age (Figure 1). Compared to the wild genotype, 25(OH)D deficiency (< 20 ng/mL) was less frequent with BsmI GA + AA (p = 0.014) and TaqI TC + CC (p = 0.034) genotypes (Figure 2). The OR for 25(OH)D deficiency among girls with the wild genotype was 1.96 (95%CI: 1.14–3.37) and 1.78 (95%CI: 1.04–3.06), for BsmI and TaqI SNPs, respectively. There was no difference in frequency of 25(OH)D deficiency between ApaI polymorphisms and the wild genotype (Figure 2) (95%CI: 0.27–1.08; p = 0.078).

In addition, taking into consideration the haplotype frequencies and the results for individual polymorphism analyses, Ht1 was considered as the risk haplotype and Ht7 as the protective haplotype for circulating 25(OH)D levels. Patients with two copies of Ht1 (Ht1/Ht1) presented lower 25(OH)D levels as compared to patients having one or no copies of Ht1 (Ht1/0 + 0/0) (19.16 ± 7.08 *vs.* 21.70 ± 6.66 ng/mL, p = 0.036) and patients having one copy of Ht1 and one copy of Ht7 (Ht1/Ht7) or two copies of Ht7 (Ht7/Ht7) (22.68 ± 6.34 ng/mL, p = 0.008), even after adjustment for season at the time of the blood collection and age.

Multiple linear regression analysis assessing the effects of season, age, BMI percentile, and polymorphic genotypes on plasma 25(OH)D are shown in Table 2. BsmI, ApaI and TaqI polymorphisms, Ht1 haplotype (GGT) and age were significant independent predictors of plasma 25(OH)D, explaining 7.4%, 4.3%, 5.6% and 4.4% of variance in plasma 25(OH)D concentrations, respectively.

**Table 2:** Multiple linear regression analysis for plasma 25(OH)D (ng/mL)

**25(OH)D *vs.* independent variables**	**Coefficient (B) ± SE**	**p**	**r^2^**
**Model 1**			
Season	−0.082 ± 1.249	0.948	0.074
Age (years)	0.469 ± 0.225	0.038	
*BMI percentile	−0.004 ± 0.015	0.804	
BsmI GG *vs.* GA + AA	3.114 ± 0.881	<0.001	
**Model 2**			
Season	−0.149 ± 1.270	0.907	0.043
Age (years)	0.518 ± 0.228	0.024	
*BMI percentile	−0.005 ± 0.016	0.739	
ApaI GG *vs.* GT + TT	2.575 ± 1.189	0.031	
**Model 3**			
Season	−0.131 ± 1.261	0.917	0.056
Age (years)	0.497 ± 0.227	0.030	
*BMI percentile	−0.005 ± 0.015	0.732	
TaqI TT *vs.* TC + CC	2.505 ± 0.890	0.005	
**Model 4**			
Season	−0.115 ± 1.268	0.928	0.044
Age (years)	0.510 ± 0.228	0.027	
*BMI percentile	−1.098 ± 1.382	0.428	
Ht1 haplotype *vs.* Ht1/0 + 0/0	2.632 ± 1.218	0.032	

* Log_10_ transformation.

## Discussion

In the present study, a high prevalence of vitamin D deficiency or insufficiency was found in 7 to 18-year old girls from South Brazil. In addition, VDR wild-type genotypes BsmI, ApaI and TaqI were associated with lower 25(OH)D levels both individually and as the GGT haplotype, even after adjustment for age and season at the time of blood collection, and were independent positive predictors of 25(OH)D levels. To the best of our knowledge, this is the first report relating VDR gene polymorphisms and haplotypes to 25(OH)D levels in healthy children and adolescent girls from the general population.

Previous studies have reported low levels of 25(OH)D in different pediatric and adolescent populations [2,3]. In our sample, only 9.4% of the participants had adequate circulating concentrations of 25(OH)D, which is far below the 39.0% reported for a 16–20 year old population from a rural town in the state of São Paulo, Brazil (latitude −23°) [17]. Since the latitude and solar radiation are similar in the cities included in those previous studies and in the cities studied by us, diverse dietary habits and the age of participants may explain the discrepancies in the frequency of sufficient 25(OH)D status. In this sense, one limitation of the present study is the lack of control for some confounders, such as dietary vitamin D intake and duration of sun exposure. However, no associations were found between 25(OH)D levels and season at the time of blood collection. One potential explanation for the lack of seasonal difference is that below a latitude of approximately 35°, UVB radiation is sufficient for vitamin D synthesis all year round [18].

Concerning young populations, several studies have shown that vitamin D levels are lower in the presence of overweight/obesity, and that low levels of 25(OH)D could influence the risk of developing metabolic disorders and cardiovascular disease in pediatric and adolescent populations [4-6]. Obesity associated-vitamin D insufficiency is likely caused by deposition of skin and dietary vitamin D3 in body fat compartments, resulting in decreased bioavailability [19].

In addition, studies have shown that obese girls with vitamin D deficiency presented lower insulin sensitivity [4], and adolescents with reduced levels of vitamin D had increased risk of metabolic syndrome [5]. Moreover, a recent study with children aged 8 to 18 years, in which 47% were obese, reported a negative association between BMI, percentage of body fat, subcutaneous and visceral adipose tissue and 25(OH)D levels in white and black subjects [3]. Despite the age similarity, the frequency of obesity was very low in our sample, and therefore we were unable to assess these associations.

Only a few studies are available in the literature relating VDR polymorphisms and 25(OH)D levels, especially in young and healthy populations. In a study with adolescent girls, the BsmI polymorphism was marginally, but not significantly, associated with 25(OH)D levels [20]. Regarding adult populations, the results are conflicting. In India, one study reported that TaqI polymorphism was associated with higher 25(OH)D levels in healthy adults aged 25 to 60 years [13], while another study analyzing BsmI and TaqI SNPs did not confirm this association [21]. In addition, other SNPs in different regions of the VDR gene have been associated with levels of 25(OH)D in adolescent girls [20] and other populations [14].

In contrast, studies focusing on specific conditions, such as the overweight Insulin Resistance Atherosclerosis family study, in which only the BsmI SNP [22] was analyzed, or the Metabolites in Multiple Sclerosis study, covering ApaI and TaqI SNPs [23], did not find an association between VDR polymorphism and 25(OH)D levels. However, these studies were carried out with disease-affected adult populations, whereas our results refer to younger, healthy subjects. In studies with older overweight subjects, BsmI, ApaI, and TaqI SNPs of VDR gene were not associated with 25(OH)D levels. Nevertheless, the lack of association may be explained, at least in part, by the fact that most of the participants in that study were using vitamin D supplements [24,25].

VDR gene polymorphisms have also been studied in some other conditions. BsmI, ApaI, and TaqI polymorphic genotypes were associated with suppressed cytokine response in pulmonary tuberculosis [26] and appear to be related with higher glucose levels [12] and diabetes [27,28]. ApaI gene variant has been linked to higher bone metabolism and bone mineral density [10]. We also have recently described that the wild genotype of the ApaI polymorphism is more frequent in girls with precocious pubarche than in controls with normal pubertal development [8].

The BsmI, ApaI and TaqI polymorphism are located at the 3’ untranslated region (3′ UTR) of the VDR gene. The BsmI and ApaI SNPs are located in intron 8, and TaqI is a silent SNP in exon 9. Although 3’ UTR has been recognized as a region involved in the modulation of gene expression, especially through the regulation of mRNA stability and efficiency of protein translation [29], the functional role of the BsmI, ApaI, and TaqI VDR gene polymorphisms has not been established. Moreover, the most common haplotype for the VDR gene in the present study (Ht1) is the same found in Caucasians and Asians, followed by Ht7 and Ht3, as found for Caucasians [30]. A strong linkage disequilibrium between these three SNPs has been shown in the present and previous studies in different populations [31].

In the present study, girls with the wild genotype for BsmI and TaqI SNPs or presenting the Ht1 haplotype were found to be at higher risk for 25(OH)D deficiency. In turn, Ht7 was regarded as the protective haplotype for circulating 25(OH)D levels. Therefore, it is possible to hypothesize that the VDR genotype influences the susceptibility to vitamin D deficiency in children and adolescents. Thus, the present results open new possibilities for further studies in this field, especially on the genotype-related mechanisms of calcium and vitamin D metabolism and prevention of 25(OH)D deficiency in young subjects.

## Conclusions

In conclusion, data from this study confirm the high prevalence of vitamin D deficiency and insufficiency in children and adolescent populations. The present findings also suggest that the BsmI, ApaI and TaqI wild variants of the VDR gene and the GGT haplotype are associated with lower 25(OH)D levels. Further studies with populations of different ethnic origins are needed to confirm the clinical relevance of the present results.

## Abbreviations

25(OH)D, 25-hydroxyvitamin D; 95%CI, 95% confidence interval; ANOVA, Analysis of variance; BMI, Body mass index; OR, Odds ratio; RFLP, Restriction fragment length polymorphism; SD, Standard deviation; VDR, Vitamin D receptor.

## Competing interests

The authors declare that they have no competing interests.

## Authors' contributions

LPGM, FS and MCSB contributed to acquisition of data, analysis and interpretation of data and manuscript review. BRS and PMS contributed to conception and study design, acquisition, analysis and interpretation of data, drafting manuscript and final review. All authors approved the final version of the manuscript.
